# Antigen-Specific Regulatory T Cells and Low Dose of IL-2 in Treatment of Type 1 Diabetes

**DOI:** 10.3389/fimmu.2015.00651

**Published:** 2016-01-11

**Authors:** Minh N. Pham, Matthias G. von Herrath, Jose Luis Vela

**Affiliations:** ^1^Novo Nordisk Research Center, Seattle, WA, USA; ^2^Pacific Northwest Diabetes Research Institute, Seattle, WA, USA

**Keywords:** low-dose interleukin-2, antigen-specific therapy, type 1 diabetes, regulatory T-cell, immune biomarker

## Abstract

Regulatory T cells (Tregs) play an important role in preventing effector T-cell (Teff) targeting of self-antigens that can lead to tissue destruction in autoimmune settings, including type 1 diabetes (T1D). Autoimmunity is caused in part by an imbalance between Teff and Tregs. Early attempts to treat with immunosuppressive agents have led to serious side effects, thus requiring a more targeted approach. Low-dose IL-2 (LD IL-2) can provide immunoregulation with few side effects by preferentially acting on Tregs to drive tolerance. The concept of LD IL-2 as a therapeutic approach is supported by data in mouse models where autoimmunity is cured and further strengthened by success in human clinical studies in hepatitis C virus-induced vasculitis, chronic graft-versus-host disease, and Alopecia areata. Treatment will require identification of a safe therapeutic window, which is a difficult task given that patients are reported to have deficient or defective IL-2 production or signaling and have experienced mild activation of NK cells and eosinophils with LD IL-2 therapy. In T1D, an LD IL-2 clinical trial concluded that Tregs can be safely expanded in humans; however, the study was not designed to address efficacy. Antigen-specific therapies have also aimed at regulation of the autoimmune response but have been filled with disappointment despite an extensive list of diverse islet antigens tested in humans. This approach could be enhanced through the addition of LD IL-2 to the antigenic treatment regimen to improve the frequency and function of antigen-specific Tregs, without global immunosuppression. Here, we will discuss the use of LD IL-2 and islet antigen to enhance antigen-specific Tregs in T1D and focus on what is known about their immunological impact, their safety, and potential efficacy, and need for better methods to identify therapeutic effectiveness.

## Introduction

Since the discovery of the 15.5 kDa protein Interleukin-2 (IL-2) numerous preclinical, clinical, and mechanistic studies have provided basic insights into its role in immunity (1–3). In 1998, Proleukin^®^ (Aldesleukin, human recombinant IL-2) was approved by the FDA for use at a sustained high dose in patients with metastatic renal cell carcinoma (MRCC) or metastatic melanoma (MM) (4, 5). The high doses and repeated administration were based on the protein’s short half-life (6). However, only a subset of treated patients exhibited improved outcome and the high-dose (HD) IL-2 treatment came with severe side effects (7, 8). Despite its long history, it is not understood why some patients respond to the therapy while most do not (7, 8). Subsequently, IL-2 has been pursued in different indications, using low doses which resulted in minimal side effects (8, 9). Studies have reported success of low-dose (LD) human recombinant IL-2 (LD IL-2) therapy in animal models of autoimmune pathology (10–14). In this context, the advantageous function of IL-2 appears to be expansion of antigen-specific regulatory T cells (Tregs) curing a number of autoimmune conditions in mice (10–14). This potential is further strengthened by success in human clinical studies in hepatitis C virus (HCV) induced vasculitis, chronic graft-versus-host disease (GVHD), T1D, systemic lupus erythematosus (SLE), and Alopecia areata (15–21).

Antigen-specific therapies in T1D aimed at regulation of the autoimmune response by tolerance induction have been filled with disappointments despite an extensive number of human trials. These trials tested primarily three autoantigens: insulin, glutamic acid decarboxylase (GAD), and heat shock protein 60 (Hsp60), either intact or components thereof (22–25). The combination of LD IL-2 together with an antigen-based therapy specific to a target organ or tissue may be a means to provide strong immunomodulation without general immunosuppression. The effect would likely involve the enhancement of antigen-specific Tregs in both their number and activity. The major question remains whether a proper dosing strategy for LD IL-2 as an induction agent can be identified that will include both an acceptable safety profile and measure of efficacy. A further consideration is how to best identify and predict efficacy of LD IL-2 therapy either with or without an antigen therapy. A dosing strategy for an antigen-based therapy is also required and the diversity of patients and the manifestation of disease must also be better understood. Currently, the common study end-points in intervention trials for T1D with LD IL-2 administration are clinical remission defined by preservation of C-peptide, decreased level of HbA1c, decreased insulin requirement, normal blood glucose, absence or reduced adverse events including vascular leak syndrome (VLS), serious infections, and allergic reactions, increased counts of eosinophils and neutrophils, and expansion of Tregs (15, 16). T1D is a complex autoimmune disease that presents in younger children in a faster, more aggressive onset, and progression. Intervention trials are mostly initiated at a later stage of disease development, when β-cell loss is well established and the intervention therapy is less likely to improve disease outcome or progression (26). Being able to conclude confidently that LD IL-2 administration is efficacious and safe for patients with T1D, we need reliable and robust immune biomarkers and assays that (1) will help to stratify the study population and (2) predict at which stage of disease progression the administration of LD IL-2 alone or in combination with an antigen-specific therapy will be efficacious in curing or preventing T1D.

## IL-2 and IL-2 Receptor

IL-2 was the first cloned interleukin in 1983, and it is arguably the most investigated interleukin with a wide role in regulation of the immune system (27, 28). Initially, IL-2 was described as a T-cell growth factor with production mainly driven by antigen-activated CD4^+^ T cells following the engagement of the T-cell receptor (TCR) and costimulation through CD28 (1, 28, 29). IL-2 is also produced, at a lower level, by activated CD8^+^ T cells, dendritic cells, natural killer (NK) cells, and NKT cells (28, 30). After its secretion, IL-2 binds to its high affinity IL-2 receptor (IL-2R) consisting of three individual subunits IL-2Rα (CD25), IL-2Rβ (CD122), and IL-2Rγ (CD132) (31). IL-2 has low binding affinity to IL-2Rα and intermediate affinity to the dimeric complex IL-2Rβ/γ (31). Analysis of the crystal structure of this quaternary complex showed that IL-2 binds first to IL-2Rα and then to the two subunits IL-2Rβ and IL-2Rγ (32–34). Naïve CD4^+^ T cells and immature and mature B-cells express almost none of the three subunits of IL-2R on their surface (28). Naïve CD8^+^ T cells, memory CD4^+^ and CD8^+^ T cells, NK, and NKT cells show low expression of the IL-2Rβ and IL-2Rγ subunits (28). Cells that express higher levels of the dimeric complex IL-2Rβ/γ are more sensitive to HD IL-2 administration resulting in severe effects including VLS (28). On the cell surface of Tregs, high expression of the tri-complex IL-2Rα/β/γ can be detected and contributes to higher sensitivity of these cells to LD IL-2 (28). Independently of the existence of IL-2Rα, both IL-2Rβ and IL-2Rγ can induce fully competent IL-2 signaling resulting in activation of phosphoinositol 3-kinase (PI 3-K)/AKT, Ras-MAP kinase, and JAK-STAT pathways for mediating cell growth, survival, death, and differentiation (2, 28, 32, 35).

**Figure 1:**
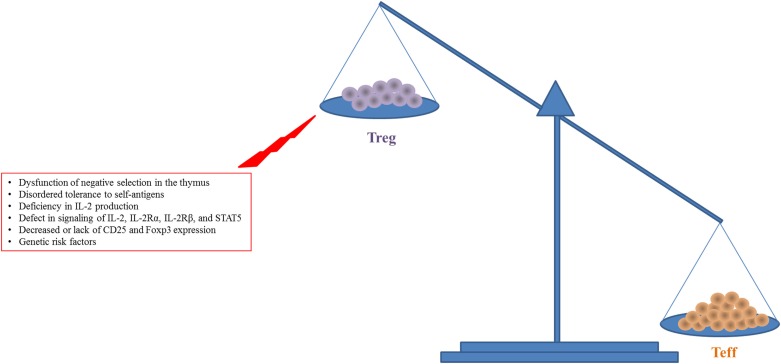
**Increased expansion and activity of Teff cells with reduced Treg number and function in autoimmune diseases like T1D are shown**. This figure simplifies a complex situation in autoimmune disease. There is a balance required for tolerance to be maintained while allowing for efficient response to pathogen or other challenges. The main insight to this balance comes from lack of Foxp3 in humans and mice where the lack of Tregs leads to unchecked destruction and death by Teff cells.

The IL-2 signal plays a major role in the homeostasis and activation of CD4^+^CD25^+^Foxp3^+^ Tregs (31). The quaternary complex consisting of IL-2 and IL-2Rα/β/γ induces the phosphorylation of STAT5, resulting in increased expression of CD25 and Foxp3 on Tregs and activation of their suppressive activity (28, 36). *In vivo* as well as *in vitro* experiments have elucidated the roles of IL-2 and IL-2R in the homeostasis and activation of Tregs (1, 37–39) (Figure 1). Deficiency in signaling through IL-2, IL-2Rα, or IL-2Rβ, or STAT5 results in lethal autoimmunity, dysregulation and decline of Treg production, and uncontrolled Teff activity (37–40) leading to an imbalance between Tregs and Teff cells (Figure 1). Patients with autoimmune disease including SLE, T1D, or rheumatoid arthritis have defective expression and regulation of IL-2 and IL-2R resulting in dysregulated Treg function and impaired downstream signaling (41–45). Data from genome-wide association studies have shown that the IL-2 locus Idd3 in non-obese diabetic (NOD) mice and 4q27 in humans are risk factors for susceptibility to development of T1D (40, 46). Boosting with exogenous IL-2 in IL-2-deficient mice led to restoration of the impaired Treg population and also prevented disease development (10–12, 14, 47). It is unclear if clinical trials have seen a similar successful remission rate with the application of IL-2 in patients with a potential risk for the IL-2-, IL-2R-, or Treg-deficiency since these were not tested (15–21). In the case of IL-2R deficiency, there would likely be no therapeutic effect with LD IL-2.

## Moving from HD IL-2 in Cancer to LD IL-2 for Autoimmune Diseases

Proleukin^®^ was considered an attractive immunotherapy for patients with MRCC or MM. The first trial to use HD IL-2 was reported in 1985 by Rosenberg et al. (48). Seven percent of patients showed complete clinical remission from MM and MRCC with 13% showing partial regression after administration with HD IL-2 (7). The most life-threatening adverse effect with HD IL-2 is VLS. This acute toxicity is mediated primarily by neutrophils and eosinophils leading to higher expression of reactive oxygen, proteases, and proinflammatory cytokines (49). Modified versions of IL-2 and IL-2 complexes are currently under development with the goal to extend the half-life of IL-2, reduce the dose of IL-2, and fine-tune the activity of IL-2 on specific target cells with the aim of reducing or eliminating severe side effects (50, 51). The vision is for an engineered “superkine” IL-2 that will only activate Tregs without activating Teff, NK cells, or eosinophils (50, 51). This is a promising direction that needs to be strongly pursued, but FDA-approved Proleukin^®^ is available now. Previous studies have shown that the administration of HD IL-2 can expand the population of Tregs in cancer patients. Subsequently, a lower dose of Proleukin^®^ was tested in patients with autoimmune disease aiming to restore the defect in Tregs (17). The first clinical trial reporting successful induction of Tregs, safety, and clinical efficacy with LD IL-2 involved patients with HCV (17). In a preclinical study, Churlaud et al. revealed that long-term administration of LD IL-2 to NOD mice neither induced systemic toxicity nor impaired the immune response (52). Nevertheless, LD IL-2 is linked to increased eosinophil and neutrophil numbers in clinical trials (15, 20, 53). Further dosing studies are required to identify the optimal dose that provides efficacy and safety in humans while limiting these side effects. This is a complex issue, given patient heterogeneity and differences in IL-2 signaling among individuals (50, 51).

## Effective Treatment of Autoimmune Disease in Murine Models with LD IL-2

In a study by Grinberg-Bleyer et al., the administration of LD IL-2 (25,000 IU) for five consecutive days resulted in remission of disease and normal blood glucose in 60% of treated NOD mice (10). The LD IL-2 induced a 1.5-fold increase in the number of Tregs in the pancreas of prediabetic mice as well as increased expression of CD25, Foxp3, CTLA-4, ICOS, and GITR which are important for Treg function have been found to be reduced in children with T1D (54). In a disease prevention study, NOD mice treated with low dose of IL-2 in the form of a IL-2/anti-IL-2 mAb complex led to a small increase in Treg cell percentage and a positive correlation with CD25 and Bcl-2 expression compared to control mice (14). No difference in expression of NK cells was reported.

Successful protection against islet β-cell destruction has also been observed with a combination therapy of rapamycin and LD IL-2 (13). Treatment with human recombinant IL-2 alone (4 ng/day) in NOD mice between the ages of 10–25 weeks did not prevent disease development while a dose-dependent decline in diabetes incidence in NOD mice treated with rapamycin alone (0.1 and 1.0 mg/kg/day) at the age of 10–33 weeks was seen. However, a synergistic effect was achieved using combination therapy with rapamycin plus human recombinant IL-2 that significantly decreased diabetes incidence in NOD mice compared to the vehicle-treated group. Disease prevention was maintained even after withdrawal of the combination therapy (13). Results like these encouraged the idea of using combinations to treat T1D, realizing the difficulty inherent in translating mouse studies to humans. We propose a LD IL-2 and antigen-specific therapy combination to induce immune tolerance occurring most likely through increase number and activity of antigen-specific Tregs. For example, a combination therapy of LD IL-2 with rapamycin has been shown to prevent allogeneic skin graft rejection in mice presumably through an increase in Treg production and decline of Teff cells detected in grafted mice (12). However, monotherapy with LD IL-2 (50,000 IU) or rapamycin did not prevent rejection (12). While rapamycin had adverse events in humans when tested in combination with LD IL-2, we propose the use of LD IL-2 in combination with an autoantigen like insulin or an insulin peptide (discussed below) be used as potential safe and efficacious therapy to prevent or reverse disease, in part by remedying the dysregulation of Treg and Teff cells, without impairing the immune response (53). The promising results in preclinical studies led to the use of LD IL-2 in the clinic. Since the therapeutic window of human recombinant IL-2 is narrow, further careful investigations are needed to determine a safe treatment strategy for LD IL-2. Evidence of the therapeutic potential of LD IL-2 in other immune diseases is compelling.

## Clinical Application of LD IL-2 in Patients with HCV

In a single-center, open-label, prospective phase 1/2 trial, Saadoun et al. investigated the efficacy and safety of low-dose Proleukin^®^ in HCV-infected patients with mixed cryoglobulinemia who were refractory to previous antiviral therapies (17). Subcutaneous (s.c.) administration of LD IL-2 (1.5 million IU/day) was administered in four courses. First, patients received a 5-day course of 1.5 million IU of LD IL-2 followed by three 5-day courses of 3 million IU/day at weeks 3, 6, and 9. Clinical improvement of HCV was seen in eight out of 10 patients after receiving LD IL-2. They did detect an increase in the percentage of CD4^+^, FOXP3^+^CD25 high (17). In a recent audit of VASCU-IL-2 trial, errors lead to a correction and update of results that include loss of significance for both the decrease of cryoglobulinemia and the increase of C4 levels during administration of LD IL-2 in original report. In addition, a grade 2 serious adverse event was discovered and included in the correction (17).

In another study with patients coinfected with HIV/HCV administration of lower dose human recombinant IL-2 led to expansion and activation of Tregs, improvement of liver function, and reduction of inflammation (55, 56). These data indicate a promising new treatment option for patients with HCV but further testing in a larger number of patients is needed (17, 55, 56). Additionally, a dose must be identified that remains efficacious with minimal or no increase in eosinophils and NK cells. Future studies should also identify long-term efficacy and identify any additional adverse events for this indication (17).

## Efficacy of LD IL-2 in Patients with GVHD

Administration of lower dose Proleukin^®^ has been shown to improve the ratio between Tregs and Teff and to induce proliferation of peripheral Tregs while enhancing their resistance to apoptosis in patients with GVHD (19). In a phase 1 study, a dose-tolerance escalation trial was conducted for LD IL-2 in GVHD patients’ refractory to glucocorticoids (20). For 8 weeks, a total of 29 patients received daily s.c. administration of LD IL-2. The patients were divided into three different dose levels (0.3 × 10^6^, 1 × 10^6^, or 3 × 10^6^ IU/m^2^ of body-surface area) (20). After a 4-week break in treatment, follow-up treatment was initiated only in responders. The dose of 1 × 10^6^ IU/m^2^ per day was the highest tolerated concentration. The higher dose of 3 × 10^6^ IU/m^2^ induced side effects that included fever, malaise, and arthralgia. In summary, there was no evidence of GVHD flare with LD IL-2 treatment, and 12 of 23 patients with GVHD showed partial response during the 8-week treatment period, with lower risk for erythema, higher joint mobility and gait, and improvement of liver function. One patient showed complete remission after 14 months even with discontinuation of both immunosuppressant and LD IL-2 (20). Additionally, two patients did not require glucocorticoid therapy after 30 and 36 months. After 4 weeks of LD IL-2 administration, the proportion of Tregs increased significantly to more than eight times the baseline level (20). However, the discontinuation of LD IL-2 led to a decline of Treg counts but remained higher than baseline. The median ratio of Treg:Teff was fivefold higher than the baseline level after 4 weeks LD IL-2 therapy. NK cells also increased during the treatment window but decreased after LD IL-2 was withdrawn. Similarly, LD IL-2 administration has been tested in patients receiving hematopoietic stem cell transplantation in order to prevent GVHD (57). No adverse events of grade 3 or 4 nor any induction of GVHD were reported. In addition to an expansion of Treg levels, viral infections were actually reduced in the LD IL-2 treated group compared to the control group (57). These data demonstrated that administration of LD IL2 was well tolerated and effective in preventing disease progression in patients with alloimmune disease. In the future, exciting clinical trials are expected for the clinical implication of LD IL-2 in GVHD (20, 57).

## LD IL-2 Therapy in Alopecia Areata

Alopecia areata is a T-cell-mediated disease involving the recognition of follicular autoantigens, resulting in inflammation-induced hair loss (58, 59). Patients with alopecia areata have a significantly higher number of Th17 cells but decreased Tregs in the peripheral blood compared to healthy controls (60). In a prospective, open pilot study, five patients with severe Alopecia areata received s.c. administration of LD IL-2 (1.5 million IU/day) for 5 days, and at weeks 3, 6, and 9 further 5-day courses of higher dose of 3 million IU/day of IL-2 (21). Using severity scoring scale for Alopecia areata, patients treated with LD IL-2 experienced a disease score improvement from 82 to 69. Furthermore, four out of five patients showed partial regrowth of scalp hair and an increased level of Tregs; however, this result was not statistically significant (60). Also, the number of recruited patients in this pilot study was very small, but the data offer promising results for a new treatment option in Alopecia areata.

**Figure 2:**
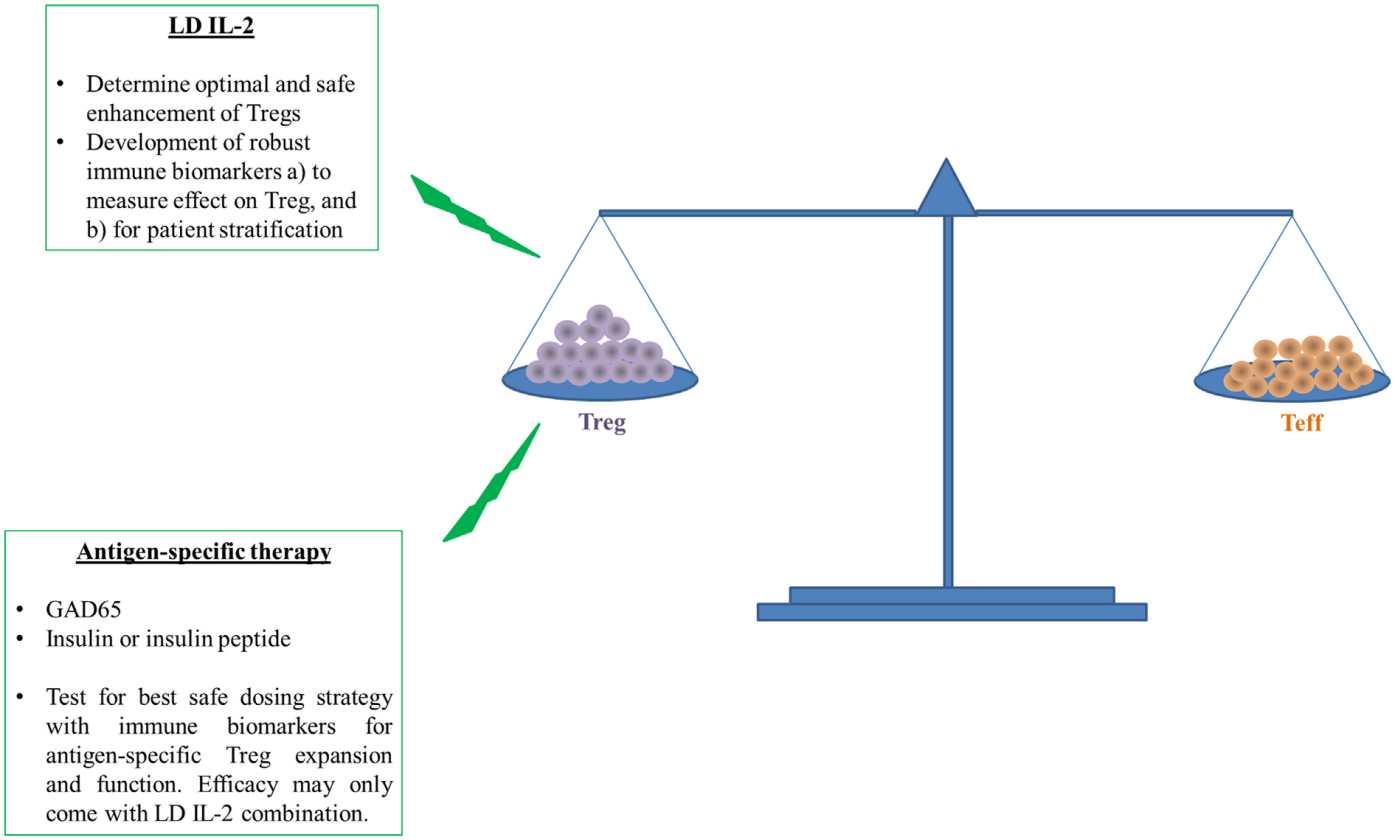
**Induction followed by maintenance of self-tolerance requires a balance between Treg and Teff to prevent or cure type 1 diabetes**. The ability of LD IL-2 to improve the number and function of Tregs to restrain auto-reactive Teffs has shown promise in humans. However, human and T1D disease heterogeneity require better measures of Treg function and overall efficacy because this therapy may be more efficacious in a subset of individuals with T1D. The overall enhancement of Treg function will likely further benefit from targeted enhancement of Treg specific for islet antigens like insulin or insulin peptide. Antigen-specific therapy requires more early validation in humans with similar need of immune biomarkers that can access therapeutic effect or patient stratification. The measure of therapeutic effect is paramount for potential testing of both LD IL-2 and antigen-specific therapy in combination.

Altogether, recent published clinical trials with LD IL-2 reported mild degrees of efficacy and safety and some evidence of restoration of impaired Treg function (17, 19–21, 57). However, it is still not clear whether the promise of LD IL-2 for treatment of autoimmune diseases will be met. Nevertheless, we need to continue progress by focusing next on the identification of the therapeutic window of IL-2, which is no easy task given the heterogeneity inherent to human populations (61, 62). Development of good and reliable immune biomarkers and assays for prediction of the clinical efficacy and possible long-term adverse effects with LD IL-2 as well as a better understanding of the mechanism of action in heterogeneous responders are therefore urgently needed (see below and Figure 2).

## LD IL-2 as a Novel Therapy for Patient with T1D

The efficacy of LD IL-2 was tested for the first time in a phase 1/2, double-blind, randomized, placebo-controlled trial with 24 T1D patients who were in an age range of 18–55 years and positive for at least one diabetes-associated autoantibody (15). The groups consisted of six patients each receiving either placebo, LD IL-2 at 0.33 MIU/day, LD-IL-2 at 1 MIU/day, or LD IL-2 at 3 MIU/day, for 5 days with a 60-day follow-up period. Overall, all patients appeared to tolerate the LD IL-2 well. The level of fasting C-peptide, incremental area under the curve (iAUC) plasma C-peptide, fasting plasma glucose, HbA1c, and insulin requirements were similar between treated groups. Significant incremental maximum effect and iAUC of Treg proportion were observed during the treatment period. A minimal but non-significant change in Teff and NK cells was also reported. Follow-up experiments tested increasing doses of LD IL-2 in an effort to determine a dose that would stimulate human Tregs without affecting other cells associated with the IL-2R (16, 62). Higher percentage of Tregs and enhanced levels of activation markers including CD25, GITR, CTLA-4, and basal pSTAT5 were detected and the data revealed that Tregs were 20-fold more sensitive to IL-2 than NK cells and memory T cells (16). In *in vitro* experiments, investigators found that increased expression of CD25 alone in activated CD4^+^ T cells did not affect the high sensitivity of Tregs to IL-2 (62). Furthermore, this study reported that the EC50 level for IL-2 induction of STAT5 phosphorylation between T1D patients and healthy subjects was similar, and there is a response gap between Tregs and CD4^+^ memory T cells in patients with T1D, which may be important in determining an effective therapeutic window for LD IL-2 for treatment of T1D (62). Additional, broader effects not directed at Tregs were also found and warrant further study and an improved understanding of Treg/Th17 balance (63) An important concern remains the potential unwanted activation of NK cells and eosinophils with LD IL-2. Overall, these studies pave the way for LD IL-2 therapy in T1D to enhance antigen-specific Tregs.

## Antigen-Specific Therapy in Patients with T1D

Autoantigens have been of interest in T1D for treating the autoimmune component of the disease coinciding with the discovery of autoantibodies and T cells specific for GAD (GAD65), insulin, insulinoma-associated protein 2 (IA2), islet-specific glucose-6-phosphatase catalytic subunit related protein (IGRP), zinc transporter 8 (ZnT8), and others (64). Many of these autoantigens were identified as targets of the damaging immune response in the pancreas and then tested in preclinical models in a setting aimed at the induction of immunological tolerance to the autoantigens. Promising results seen in previous preclinical studies for preventing or reversal of T1D led to clinical trials (24, 65–69). Given the evidence in preclinical models, it is of great interest to test the efficacy of antigen-specific therapy in combination with LD IL-2. Unfortunately, most clinical trials testing autoantigens to prevent or cure T1D were disappointing. The trials focused primarily on three autoantigens insulin, GAD, and Hsp60, administered as intact protein or peptide derived from the autoantigen with very different routes of administration. In the case of insulin, the results have not indicated any significant effect despite attempts at the delivery of insulin orally, intranasally, and subcutaneously (24, 67, 68). A closer inspection of these disappointing results is important given the lack of information on optimal dosing strategy or route used in each trial. In the case of oral antigen administration, doses required in mice are larger than any used in clinical trial, and it is unclear how much insulin would remain undigested by the time it reached the small intestine without any encapsulation (70). The hsp60 peptide Diapep277 had marginal reduction in C-peptide and GAD65 in alum slowed beta cell loss, but the results were not repeated in Phase III (71–75). However, valuable information was obtained from these studies, including evidence of increased IL-10 and reduced T cell responses in patients treated with Hsp60, while patients treated with GAD alum had increased Tregs, T-cell proliferation, and inflammatory cytokines (76). It is imperative that we find appropriate immune biomarkers of response to antigenic therapy that measure immune responses to therapeutic self-antigens early during treatment (briefly mentioned below). The most promising antigen candidate for this therapy is insulin or an insulin-derived peptide. The basic idea would be to induce an infectious tolerance with the insulin-derived autoantigen that would provide protection from immune responses against other known T1D islet antigens. For example, it would be highly important if the number and function of Tregs specific for insulin could accurately and reliably be measured during and after treatment with the self-antigen. We believe that antigen therapy may not work alone but is better suited for use in combination with LD IL-2 or another induction agent and the antigen will likely have to be given continuously or as booster. Further studies are needed in both mice and human to validate the proposed combination in T1D. In addition, we must seriously consider the use of combination therapies that also work on metabolic dysregulation in the islets since there are likely components of T1D that are not immune related that could be of greater benefit if combined in the future (26). Biomarkers will have to be developed to better understand the natural history of T1D and that will inform clinical trials of the action or efficacy of a particular therapy.

## To Move the Field Forward: Reliable and Robust Immune Biomarkers

The path toward a cure in T1D has been filled with many challenges (25, 64, 65). Many clinical trials have tested potential therapies for a cure or delay in the onset of T1D (24, 65–73, 77). However, the promising results seen in the NOD mouse model have, thus far, not translated to the clinic (24, 65–73). Unfortunately, reasons for failures are poorly understood in large part due to our lack of understanding about the cause or natural history of T1D and the difficulty of enrollment of younger age groups, where therapy may be more efficacious, but ethical and regulatory body restrictions limit or prevent participation.

In clinical trials, the C-peptide level has been relied on as a clinical endpoint together with blood glucose, HbA1c, and insulin requirement. C-peptide provides information about β-cell function and insulin production, but it is heterogeneous among patients depending on age, stage of diagnosis, disease progress, and sex with a slow decline in adults compared to children (26, 78, 79). For this reason, a more sensitive immune biomarker is required that could also allow for stratification of patients enrolling in clinical trials. From previous trials, it has been reported that higher C-peptide level is associated with lower HbA1c, reduced insulin requirement, reduced hypoglycemic events, and reduced retinopathy (80). Better stratification of patients would require immune biomarkers that could follow TID natural history (81, 82). Additional surrogate markers of β cells are needed to identify cell destruction, stress, or metabolic imbalance not limited to immune system parameters. Examples include miRNA, methods to measure pancreas size, or composition, and non-invasive imaging (26).

T1D is in part an immune-mediated and antigen-driven disease resulting in an imbalance between Tregs and Teff, we therefore need validated immune biomarkers and assays that can give us clear answers (1) about the functionality and quantity of islet antigen-specific T cells; (2) whether the intervention therapy will enhance Tregs; and (3) whether the restoration of antigen-specific or polyclonal Treg number and function will correlate positively with the pancreatic islet function.

Previous studies have reported that the disease development of T1D is associated with the presence of a specific signature of cytokines and adhesion molecules that can be captured by ELISA or Luminex assays (83). However, the organ that suffers most during the disease development of T1D is the pancreas; therefore, we need robust methods that can measure the activity and infiltration of immune cells in the pancreas and overall size and composition.

In the clinic, it is common to test for the existence of diabetes-associated autoantibodies, including GAD65, IAA, IA2, IGRP, and ZnT8 for diagnosis of patients with T1D (83, 84). It has been discussed that the titer and number of autoantibodies are a useful biomarker to predict disease progress in patients with T1D (84, 85). Currently, assays for measuring antigen-specific CD8 or CD4 T cells in both freshly isolated and cryopreserved peripheral blood mononuclear cells samples with flow cytometry-based HLA multimers are in progress and have been validated (84). Unfortunately, the percentage of autoantigen-specific T cells in the periphery is very low and often cannot be detected (84). Also, new barcoding with quantum dots has enhanced detection of autoreactive T cells but still require improvement (26, 84). In clinical trials, ELISpot is a common and widely used method to provide information about the functionality and quantity of immune cells (84–86). With ELISpot assays, we can measure cytokine responses to islet-specific antigens in T1D patients and healthy subjects (84). The number of cytokines that can be detected is increasing with improved fluorospot assays but still require development (84).

The failure for previous clinical trials may have not been only related to deficiencies in dosing and administration of tested therapies but also confounded by the late initiation of intervention therapy in patients. As T1D development is more prevalent in children and their disease progression more aggressive than in adults, we may also consider the effect of age in conducting a successful intervention trial. Some therapies may be more effective in children than in adult patients (26). However, we still do not know the exact window of opportunity for treatment in humans, and the therapy has to first be tested and proven safe in adults before given to children. To achieve a successful and effective intervention study, we need a better strategy that includes stratification for study participants for future clinical trials.

Given the promising data of LD IL-2 in patients with HCV, GVHD, and Alopecia areata, there is great interest in giving LD IL-2 to patients with T1D to specifically boost Treg numbers and activity (15, 16, 62). This treatment can then be enhanced with an antigen-based therapy to increase the number and activity of Tregs in patients with T1D. In order to make progress with the application of LD IL-2 with an antigen-mediated therapy in clinical trials, it is required to develop robust immune biomarker assays to determine the extent of expansion of Tregs in quantity and quality that can help stratify patients, tell us whether a given therapeutic hit the right pathway. Most importantly, we must develop biomarkers that teach us about the natural history of the disease. These reliable immune biomarkers will enable optimal clinical study plans and enrollment of the proper cohorts of patients, regardless of age, that would benefit most from LD IL-2 and antigen-based therapy. For more focus on biomarkers, please see Ryden et al. (77).

## Summary

In the present review, we discuss recent reports describing efficacy and side effects with LD IL-2 treatment in preclinical and clinical experiments of select autoimmune diseases. A common theme emerges: there is a mild increase and expansion of Tregs observed in murine models as wells as in patients. However, a slight increase of NK cells has been reported in some clinical trials even using lower dose of Proleukin^®^. Our need for a better and safer dosing strategy of low dose of Proleukin^®^ is required for efficient and safe use in T1D and other autoimmune diseases (Figure 2). Importantly, biomarkers are needed to determine the safe expansion of Tregs in quantity and quality. The goal of developing robust immune biomarkers will help us to stratify and treat the patients in whom we know LD IL-2 therapy will be efficacious (Figure 2). At the moment, the lack of robust and reproducible immune biomarkers is a bottleneck. Further effort will be needed to make the treatment of LD IL-2 safer and more efficacious.

## Author Contributions

MP, JV, and MH contributed to concepts of the work, drafting, revising, and final approval of the review article.

## Conflict of Interest Statement

The authors are affiliated with (Minh N. Pham) or work for (Jose Luis Vela and Matthias G. von Herrath) Novo Nordisk, Inc.
